# Primary Pyomyositis: Contact Sports as the Rare Risk Factors

**DOI:** 10.1155/2019/5739714

**Published:** 2019-07-28

**Authors:** Behzad Amoozgar, Varun Kaushal, Bhaveshkumar Garsondiya

**Affiliations:** Jersey Shore University Medical Center, Perth Amboy Division, Perth Amboy, NJ, USA

## Abstract

Primary pyomyositis is an infectious disorder that mostly involves children and adults. Direct injury to the muscle or any traumatic process that can cause bacteremia has been described as the common risk factor. Contact sports without direct contusion or injury to the muscle is an uncommon culprit for the manifestation of this disease. In our case, a young male athlete presented to the emergency room with vague signs and symptoms including right leg muscle pain and fever. He denied any direct injury or contusion of the muscle. CT scan was done and showed edematous gluteus minimus muscle. MRI as one of the best tools for investigating soft tissues was done and exhibited myositis. Blood culture became positive for the methicillin-susceptible *Staphylococcus aureus*. Appropriate antibiotics were started, and the patient condition was improved. Considering prominent risk factors, early diagnosis and treatment of pyomyositis are major key factors for the management of these infectious conditions as it may cause loss of the limb or even result in mortality.

## 1. Introduction

Pyomyositis is a bacterial infection of skeletal muscles. Classically, it is an infection found mostly in the tropics. It affects two age groups which are 2- to 5-year-old children and 20- to 45-year-old adults, with higher prevalence in men. Predisposing factors for pyomyositis are trauma, concurrent infections, and malnutrition. Pyomyositis has been described among athletes performing vigorous exercise, suggesting a potential role of muscle damage in the pathogenesis of the disease.

## 2. Case Description

An 18-year-old male high school wrestler with no significant past medical history came to the emergency department (ER) because of cramping right leg pain. The pain started one day after wrestling which he denied any direct below (no history of trauma) to the area or any penetrating injury during the activity.

In ER, the patient was found to have a fever of 101.9 along with weakness in the right leg. During the examination, swelling and tenderness over the lateral right leg were evident. The patient was admitted, and workup was initiated including X-ray and CT scan of the right leg and blood cultures. The erythrocyte sedimentation rate was 25 (1–20 normal range), and CRP was 13 (<0.5 normal range).

X-ray was negative, and the CT scan showed slight edema involving the right gluteus minimus muscle. Two blood cultures were sent which came back positive for methicillin-susceptible *Staphylococcus aureus* or MSSA. We started vancomycin IV, and infectious disease (ID) was consulted as no known source of infection was found. ID requested MRI.

**Figure 1:**
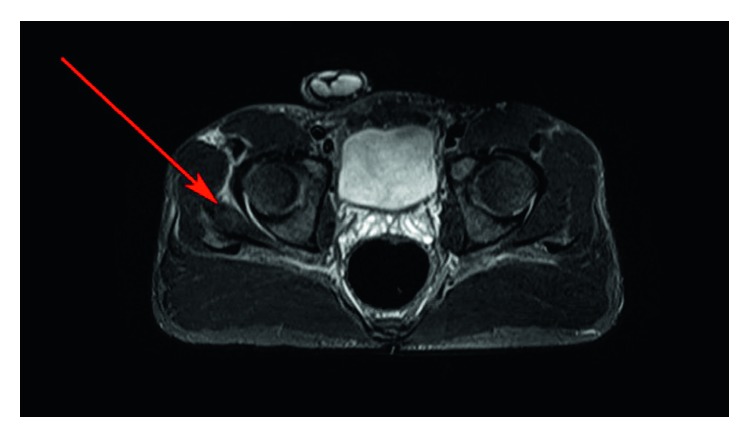
MRI (red arrow) shows moderate intramuscular edema at the ventral aspect of the right gluteus minimus muscle with mild muscle enhancement.

MRI results exhibited mild soft tissue edema in between the right iliac bone and the right gluteus minimus muscle, which extended laterally to the superficial aspect of the right proximal rectus femoris muscle and inferiorly to the superficial and deep portion of the gluteus medius muscle at its greater trochanteric insertion. The MRI report suggested myositis (Figure 1) of the right gluteus minimus muscle. Additionally, MRI showed reactive bone marrow edema of the right iliac bone, abutting the right gluteus minimus muscle.

As per ID request, interventional radiology (IR) was consulted and the case was discussed with an orthopedic surgeon and general surgeon on service. ID and IR agreed to proceed with ultrasonography joint arthrogram. IR-guided fluoroscopy was performed for possible aspiration of the joint and rule out septic arthritis. Successful access of the right hip synovial space was done, but no significant effusion was found for drainage. No further intervention was recommended by IR, ID, orthopedic surgeon, and general surgeon.

Next two sets of blood culture came back positive for MSSA. The patient was switched to nafcillin based on sensitivities. Repeat cultures became negative. Further workup included a transesophageal echo that showed no endocarditis. The patient condition improved and was discharged with PICC line and ceftriaxone to be received at medical day stay for four weeks.

## 3. Discussion

Pyomyositis is a purulent infection of skeletal muscle that usually manifests with abscess formation [1].

*Staphylococcus aureus* is the most common organism isolated [2, 3].

Pyomyositis is divided into 3 clinical stages with the first stage having crampy muscle pain, mild fever, and mild leukocytosis. The second stage shows the patients with worsening muscle pain, edema, and abscess formation. Lastly, the third stage with a person showing signs of systemic toxicity such as septic shock or endocarditis. In our case, the progression of the disease was limited due to early intervention.

Although pyomyositis is more prevalent in tropical countries, recent studies have shown that this disorder is no longer restricted to tropical regions [4]. Furthermore, recent articles have shown that this entity is not limited to immunocompetent patients [5]. MRI is a promising modality for the detection of this disorder and exclusion of other diseases such as septic arthritis [6].

Common known risk factors for pyomyositis are immunodeficiency, [7] trauma, concurrent infections, and malnutrition. Recently, it has been proposed that strenuous exercise may be a possible risk factor. However, it is hard to justify how excessive exercise may lead to bacteremia which is the most common pathophysiology suggested for this disorder. In our case, the patient participated in wrestling activity. Hypothesized by the previous study, participating in a contact sport, which necessitates coming into contact with body part/muscle contacts and manipulations, needs to be considered as a rare but potential risk factor [8].

The incident of pyomyositis is increasing in temperate countries. This is a rare case in the New Jersey area where pyomyositis was established after contact sport. Though not common in the United States of America, a patient presenting to ER with high risk of trauma or traumatic episode while having elevated fever and bacteremia and pyomyositis should be kept in mind.

## 4. Conclusion

The patient is an 18-year-old high school wrestler presenting with pyomyositis as a likely result of vigorous exercise during contact sport and possible minor nonevident trauma. All common and uncommon risk factors should be considered during the clinical approach.
